# Is the Random Forest Algorithm Suitable for Predicting Parkinson’s Disease with Mild Cognitive Impairment out of Parkinson’s Disease with Normal Cognition?

**DOI:** 10.3390/ijerph17072594

**Published:** 2020-04-10

**Authors:** Haewon Byeon

**Affiliations:** Department of Speech Language Pathology, School of Public Health, Honam University, Gwangju 62399, Korea; bhwpuma@naver.com

**Keywords:** cognitive function, data mining, Parkinson’s disease with mild cognitive impairment, random forest, neuropsychological test

## Abstract

Because it is possible to delay the progression of dementia if it is detected and treated in an early stage, identifying mild cognitive impairment (MCI) is an important primary goal of dementia treatment. The objectives of this study were to develop a random forest-based Parkinson’s disease with mild cognitive impairment (PD-MCI) prediction model considering health behaviors, environmental factors, medical history, physical functions, depression, and cognitive functions using the Parkinson’s Dementia Clinical Epidemiology Data (a national survey conducted by the Korea Centers for Disease Control and Prevention) and to compare the prediction accuracy of our model with those of decision tree and multiple logistic regression models. We analyzed 96 subjects (PD-MCI = 45; Parkinson’s disease with normal cognition (PD-NC) = 51 subjects). The prediction accuracy of the model was calculated using the overall accuracy, sensitivity, and specificity. Based on the random forest analysis, the major risk factors of PD-MCI were, in descending order of magnitude, Clinical Dementia Rating (CDR) sum of boxes, Untitled Parkinson’s Disease Rating (UPDRS) motor score, the Korean Mini Mental State Examination (K-MMSE) total score, and the K- Korean Montreal Cognitive Assessment (K-MoCA) total score. The random forest method achieved a higher sensitivity than the decision tree model. Thus, it is advisable to develop a protocol to easily identify early stage PDD based on the PD-MCI prediction model developed in this study, in order to establish individualized monitoring to track high-risk groups.

## 1. Introduction

Over the past decade, the field of geriatrics has experienced emerging interest in Parkinson’s disease with mild cognitive impairment (PD-MCI) [1,2,3,4]. The Sydney cohort study [5], the most highly representative epidemiology study on the subject, examined 136 patients diagnosed with Parkinson’s disease (PD) over 20 years. The study reported that 84% of PD patients had cognitive impairment, and 50% of them progressed to PD dementia (PDD). Likewise, PD is often accompanied by cognitive dysfunction in addition to dyskinesia [2].

The mild cognitive impairment (MCI) stage is the earliest at which we can detect dementia [6]. Because it is possible to delay the progression of dementia when it is detected and treated in an early stage, identifying MCI is an important primary goal of dementia treatment [6]. PD-MCI is frequently found in patients with PD [7,8]. However, the sociodemographic and neuropsychological characteristics of PD-MCI are less well-known than those of MCI and vascular mild cognitive impairment (vascular-MCI) [7,8]. The distinctive neuropsychological characteristics found in early stage PD-MCI are caused by executive function damage due to prefrontal hypofunction or malfunction [9]. However, it is difficult to distinguish PD-MCI from MCI or vascular-MCI, because they show similar symptoms [10]. Additionally, people with PD experience a slowly deteriorating cognitive deficit and impaired motor function, which can be mistaken for cognitive frailty as part of the normal aging process. As a result, it is difficult to diagnose early stage PD. MCI can be diagnosed based on interviewing, cognitive function evaluation via a standardized neuropsychological test, and brain imaging such as magnetic resonance imaging (MRI). It is possible to diagnose cerebrovascular diseases or to analyze brain atrophy using brain imaging. However, this is unsuitable for early PD diagnosis, because brain atrophy can be confirmed visually only at a very advanced stage. Therefore, neuropsychological testing that also tests cognitive function has been used as an effective screening test for diagnosing MCI [11].

Recent studies have pointed to the necessity of considering mental health, such as depression, while diagnosing MCI [12,13]. In particular, the development pattern and risk factors of cognitive impairment are known to vary according to race. Therefore, it is necessary to develop an MCI prediction model reflecting the characteristics of the neuropsychological indices and lifestyles of the elderly in South Korea; however, South Korea has less systematic epidemiological data on cognitive impairment in the elderly than other countries such as the United States and European countries. In South Korea, previous community-based epidemiological studies on PD have been conducted on patients living in a single city [14]. However, there has been no study to develop a prediction model based on a nationwide epidemiological survey. Moreover, most of the previous studies [15,16] evaluating the neuropsychological characteristics of patients with PD have used regression models. Regression models are effective in exploring the neuropsychological characteristics of individual risk factors but are limited in analyzing multiple risk factors simultaneously. It is also difficult to prioritize risk factors with regression models. Linear regression models in particular require several assumptions, including linearity, equal variance, and a normal distribution, but disease data have been known to violate these assumptions.

In recent years, the medical field has applied data mining to predict the risk of diseases and vulnerable groups [16,17]. Data mining is a type of big data analysis that examines the relationships and rules within a dataset to extract valuable information [18]. The health science field has traditionally used tree-based methods such as Classification and Regression Tree (CART) as data mining methods for disease prediction [19]. Decision trees carry the risk of overfitting, and the accuracy of decision trees can vary greatly depending on the training data (input variables). Random forests, a data mining method developed in 2001, were designed to overcome these limitations. Random forests generate multiple decision trees by conducting random sampling on the same dataset and combining them to predict the target variable. Therefore, the accuracy of random forests is higher than that of decision trees [20,21]. Moreover, random forests can be used to explore the relationship between explanatory variables and diseases when many (types of) explanatory variables are applied to a random forest model [22]. In addition, the prediction power of random forests outperforms the bagging model [22].

Several previous studies [23,24,25] have reported on Parkinson’s dementia predictors using biomarkers such as cerebrospinal fluid (CSF) and electroencephalogram (EEG) data. However, we are unaware of any study that identifies the predictors of PD-MCI for patients with PD and normal cognition (PD-NC), taking into account sociodemographic factors, lifestyles, depression, and neuropsychological characteristics. The objectives of this study were to develop a random forest-based PD-MCI prediction model considering health behaviors, environmental factors, medical history, physical functions, depression, and cognitive functions by using the Parkinson’s Dementia Clinical Epidemiology Data (a national survey conducted by the Korea Centers for Disease Control and Prevention), and to compare its prediction of accuracy with those of decision tree and multiple logistic regression models.

## 2. Methods

### 2.1. Data Source

This study was conducted using the Parkinson’s Dementia Clinical Epidemiology Data obtained from the National Biobank of Korea, the Center for Disease Control and Prevention, the Republic of Korea (no. KBN-2019-005). We obtained the approval of the Research Ethics Review Board, the National Biobank of Korea (no. KBN-2019-005), and the data use approval of the Korea Centers for Disease Control and Prevention (no. KBN-2019-1327). The National Biobank of Korea was established in 2008 with the approval of the Ministry of Health and Welfare and is managed by the Korea Centers for Disease Control and Prevention for the emerging necessity of managing bio-data systematically at a national level. The ultimate goal of the National Biobank of Korea is to promote biomedical research and public health. Please refer to Lee et al. [26] for the specific activities of the National Biobank of Korea, including its quality control programs. 

The Parkinson’s Dementia Clinical Epidemiology Data used in this study were collected under the supervision of the Korea Centers for Disease Control and Prevention at 14 tertiary care organizations (university hospitals) from January to December 2015. Health surveys, including health behavior questions, were conducted using computer-assisted personal interviews. The data are composed of sociodemographic factors (e.g., gender), environmental factors (e.g., exposure to pesticides), health behaviors (e.g., smoking), disease history (e.g., hypertension), exercise characteristics related to PD (e.g., tremor), sleep behavior disorders (e.g., rapid eye movement (REM)), and neuropsychological characteristics (e.g., cognitive function). PD-MCI was diagnosed by neuropsychologists according to the criteria of the International Working Group on MCI [27].

### 2.2. Subjects

**Figure 1:**
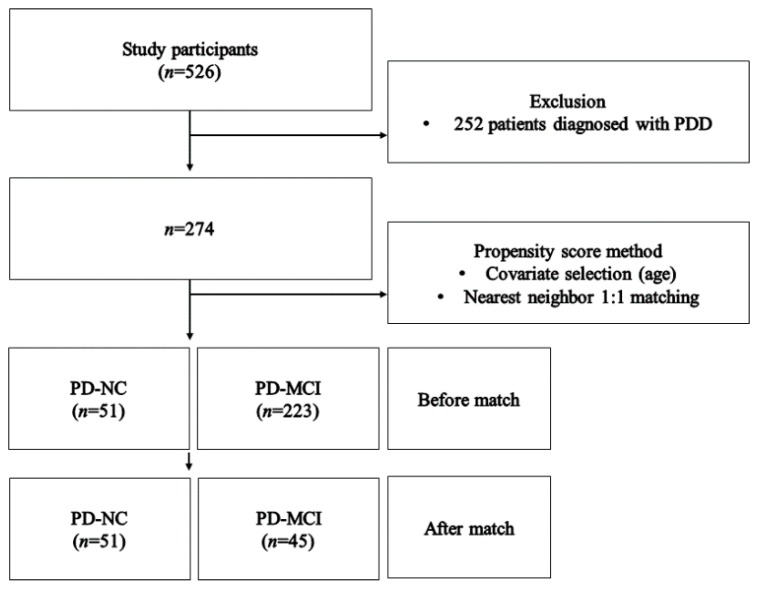
Framework of study.

Observational studies frequently utilize secondary data and these studies are more likely to experience data imbalance while comparing patients and healthy subjects [28]. Propensity score matching (PSM) was used to minimize selection bias and resolve the imbalance of case-control [29]. This study found an imbalance between PD-NC and PD-MCI. In order to solve this issue, this study used PSM, balancing between populations using the nearest neighbor matching by controlling the age of the case-control group [30]. Moreover, this study excluded individuals (subjects) that did not match in both groups in common to ensure good data balance. Before matching, there were 274 subjects (PD-MCI = 223; PD-NC = 51), and, after conducting PSM, it was matched to 96 subjects (PD-MCI = 45, PD-NC = 51; Figure 1). This study finally analyzed 96 subjects.

### 2.3. Measurement

The outcome variable is defined as the prevalence of PD-MCI classified by medical diagnosis. The explanatory variables included age (60–74 years old or ≥75 years old), gender (male or female), education (middle school graduate and below, or high school graduate and above), handedness (left hand, right hand, or both hands), family dementia history (yes or no), family PD history (yes or no), pack-years (non-smoking, 1–20, 21–40, 41–60, or ≥61 pack-years), coffee-drinking (yes or no), mean coffee intake per day (no, ≤1, 2–3, or ≥4 cups), coffee drinking period (no, ≤5, 6–9, or ≥10 years), pesticide exposure (never, currently not exposed but exposed previously, or currently exposed to pesticide), disease history (carbon monoxide poisoning, manganese poisoning, encephalitis, traumatic brain injury, stroke, alcoholism, diabetes, hypertension, hyperlipidemia, and/or atrial fibrillation), PD related motor signs (tremor, akinesia/bradykinesia, postural instability, and/or late motor complications), REM, sleep behavior disorders, neuropsychological characteristics such as those outlined in the Korean Mini Mental State Examination (K-MMSE) [31], Korean Montreal Cognitive Assessment (K-MoCA) [32], Geriatric Depression Score (GDS) [33], global Clinical Dementia Rating (CDR) score [34], Korean Instrumental Activities of Daily Living (K-IADL) score [35], Untitled Parkinson’s Disease Rating (UPDRS) total score [36], UPDRS motor score [37], Hoehn and Yahr staging (H&Y staging) [38], and the Schwab and England Activities of Daily Living scale (Schwab and England ADL) [39]. These variables are defined in Table 1.

### 2.4. Development and Evaluation of Prediction Models

The prediction model was developed using a random forest algorithm, and the results of the developed prediction model were compared with those of a decision tree based on multiple logistic regression and a classification and regression tree. The prediction accuracy of the model was calculated using the recognition rate.

**Figure 2:**
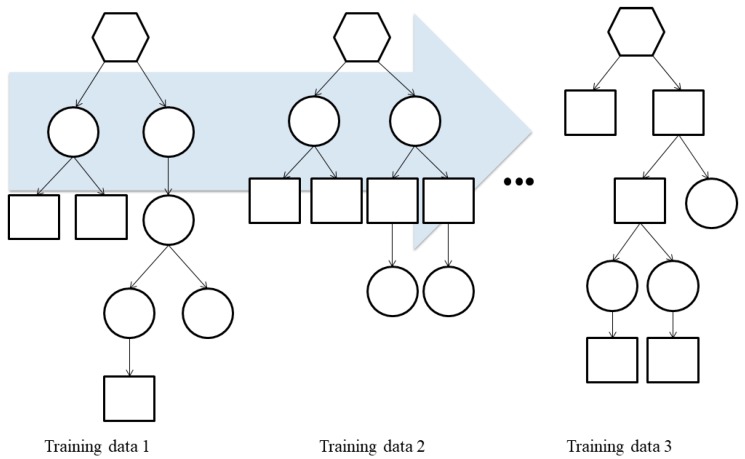
Ensemble classifiers that combines many single decision trees.

Random forests are ensemble classifiers that randomly learn multiple decision trees. The random forest method consists of a training step that constructs several decision trees, and a test step that classifies or predicts an outcome variable based on an input vector. The ensemble form of random forest training data can be expressed as Forest F = {f*1*, ..., f*n*} (Figure 2). The distributions obtained from the decision trees of each forest were first averaged by T (the number of the decision trees) and then classification was conducted. The predictors of each sample were combined by using the mean for continuous target variables and the majority vote for categorical target variables.

(1) $$L\left(p\right)=\frac{1}{T}\;\overset{T}{\underset{t=1}{\sum }}P_{t}\left(b|I,\;p\right)$$

Random forest is similar to the bagging technique, because both approaches combine decision trees generated from multiple bootstrap samples using the majority vote principle in order to increase stability. However, they are different, because the former uses a few explanatory variables that were randomly selected from each bootstrap sample. 

This study presented a partial dependence plot and variable importance to show the prediction power of the main explanatory variables. The variable importance indicates the effect of an explanatory variable on the accuracy of a model. Therefore, when an explanatory variable improves the performance of a model, the importance of the variable increases. A partial dependence plot shows the changes in response variables according to the continuous change of each explanatory variable. The contribution of a dependent variable to an independent variable is expressed as a function of a variable. The function of partial dependence is presented in Equation (2).

(2) $$\left(\frac{p_{1}\left(x,\;x_{ic}\right)}{p_{0}\left(x,x_{ic}\right)}\right)$$

RF can be free from overfitting theoretically, and is not affected by noise or outliers much [20]. Moreover, it can generate high accuracy results by reducing generalization errors [20]. However, RF is more likely to have an elbow point, which means a steep drop in slope with more trees. Moreover, there is a higher probability that each tree will be more complex when an unimportant explanatory variable is selected. Therefore, this study improved the accuracy of the model by considering the number of mtry, the number of candidate explanatory variables, in advance.

The prediction performance of a model was validated while considering the overall accuracy, sensitivity, and specificity together. Sensitivity means the prediction accuracy of PD-MCI, while specificity indicates that of PD-NC. As the objective of this study was to develop a model that can predict PD-MCI, this study considered overall prediction accuracy and sensitivity as the most important factors for evaluating prediction performance. When the overall prediction accuracies and sensitivities of the two models were identical, their specificities were compared. This study first established a random forest model and then compared the results and the accuracies of models obtained from multiple logistic regression and CART. In this case, forward selection based on standard likelihood ratio tests was used to select variables in the multiple logistic regression analysis. All of the statistical analyses were conducted using the “RandomForest” package of R-version-3.6.1 (Foundation for Statistical Computing, Vienna, Austria).

## 3. Results

### 3.1. General Characteristics of the Subjects

The General characteristics of the subjects are presented in Table 2. Of the 96 subjects (after match), 47.9% were male, 52.1% were female, 38.5% had a high school or above level of education, 8.0% had a family history of PD, and 6.8% had a family history of Alzheimer’s dementia. Additionally, 5.7%, 2.3%, 23.2%, and 40.0% of the subjects had a history of head injury (e.g., traumatic brain injury), stroke, diabetes, and hypertension, respectively.

### 3.2. Major Risk Factors of Random Forest-Based PD-MCI Prediction Model

**Figure 3:**
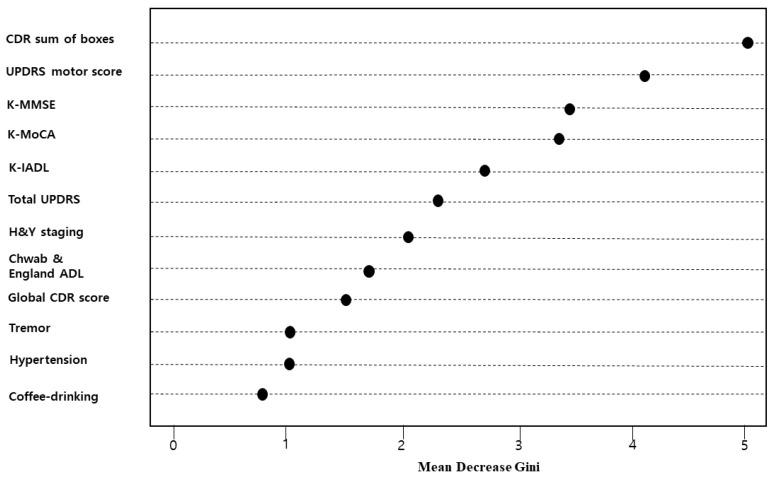
Variable importance in a random forest model (showing only the top 12 factors).

A PD-MCI prediction model was established using random forests, and the results are presented in Figure 3. Some of the random forest models estimated major risk factors using decreased in the GINI coefficient. The major risk factors of PD-MCI were, in descending order of magnitude, CDR sum of boxes, UPDRS motor score, the K-MMSE total score, and the K-MoCA total score. Among these factors, the UPDRS motor score was the most important predictor of PD-MCI. In contrast, the importance of atrial fibrillation and stroke was zero.

**Figure 4:**
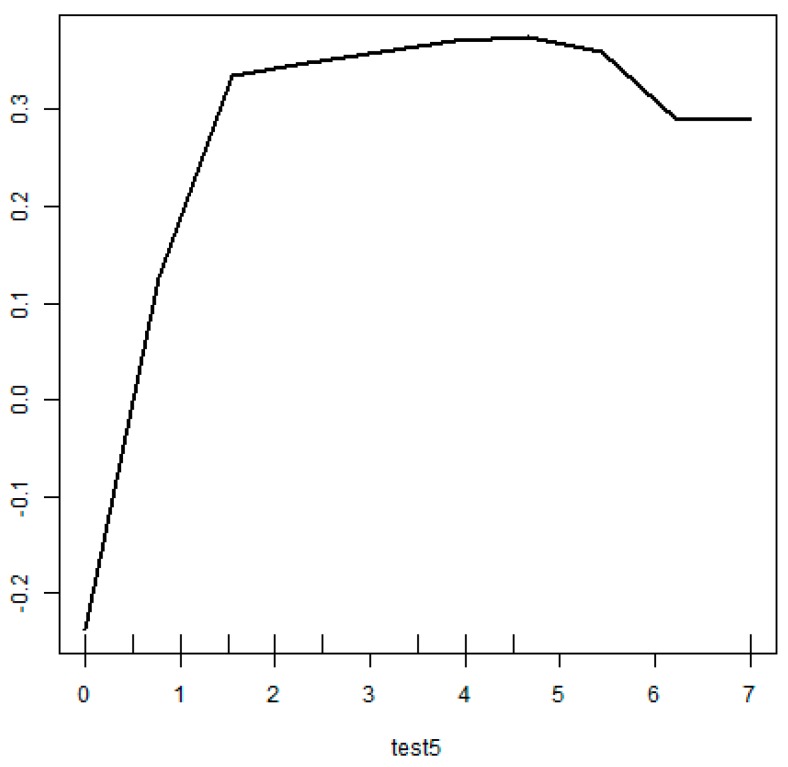
Partial dependence plot (CDR sum of boxes).

The partial dependence plot regarding the CDR sum of boxes, the most important variable in the predictive model, is presented in Figure 4. The results showed that, when other factors were constant, the risk of PD-MCI increased with a higher CDR sum of boxes (Figure 4).

### 3.3. Comparison of the Accuracy of the Developed Prediction Models

This study changed the mtry values (numbers), presenting the number of explanatory variables to be used in the decision tree constituting RF, from 5 to 15, and selected the value with the smallest error of Out-Of-Bag. The changes in the error of Out-Of-Bag are presented in Table 3. The optimal mtry to be applied in this study was 5, showing the lowest error rate (34.4%). 

**Figure 5:**
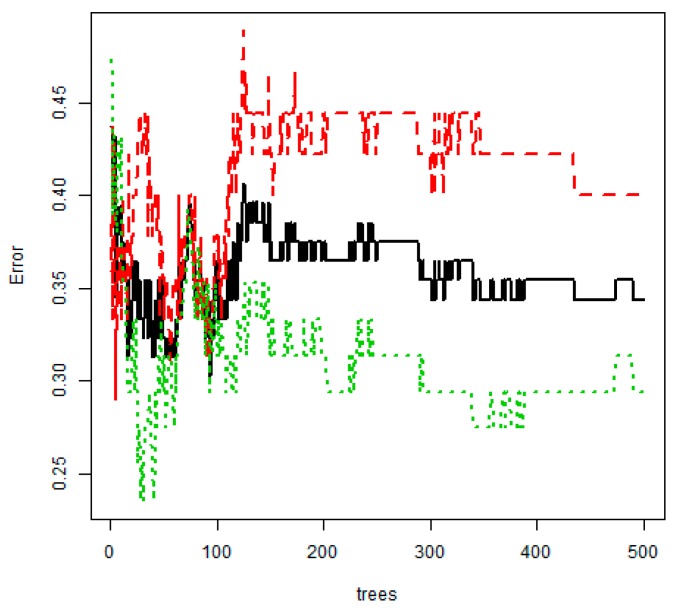
Out-of-bag error rate curve (random forest model). Black line—overall accuracy; red line—sensitivity; Green line—specificity.

When ntree, the number of tree generation, and mtry were set as 500 and 5, respectively, the final RF model of this study had an overall accuracy of 65.6%, a sensitivity of 70.6%, and a specificity of 60.0% (Table 4). On the other hand, the overall accuracy of CART was calculated as 67.7%, higher than that of RF, but the sensitivity of it was the lowest (51.1%). In Figure 4, the black line indicates the changes in each error rate against 500 bootstrap samples. Figure 5 shows that the changes in error rate become relatively stable after the number of bootstrap samples exceeded 150.

## 4. Discussion

Diagnosing early stage PD-MCI is important in the health sciences, because it can delay the cognitive decline associated with PDD. Previous studies [22,40] have reported that the impairment of the executive function is a major cognitive feature of PDD. However, it is challenging to distinguish PD-MCI from PD-NC solely based on executive function. Therefore, we explored the major differential indicators of PD-MCI, taking into account sociodemographic variables, health habits, PD related motor and non-motor symptoms, cognitive tests, and neuropsychological tests. We developed a PD-MIC prediction model based on random forests, and confirmed that the CDR sum of boxes, UPDRS motor score, K-MMSE total score, and the K-MoCA total score were major predictors of PD-MCI. Among all of the neuropsychological screening tests, the CDR sum of boxes was the most important predictor for distinguishing PD-MCI from PD-NC. Therefore, when a neuropsychological test is performed to diagnose PD-MCI in patients with PD, the CDR (sum of boxes) scoring should be conducted first over other cognitive-language screening tests so as to achieve higher sensitivity. 

Previous studies [41,42] examining the sociodemographic and emotional characteristics of PDD reported that depression is the main characteristic of PDD. For example, Aarsland et al. (2007) [41] evaluated 537 patients with PDD and observed that 58% of the patients had depression. However, in the present study, depression was not an important indicator for predicting PD-MCI. This might differ from previous studies [41,42], because previous studies compared healthy elderly individuals versus those with PD-MCI, while the present study only examined people with PD. In other words, depression is potentially not a major differential indicator in this study, because both PD and PD-MCI have high depression rates (31.3%). As only a few studies have tried to distinguish PD-MCI from PD-NC considering neuropsychological characteristics, health habits, and depression, more observation studies on PD-MCI are needed in order to verify the major predictors of PD-MCI.

Another meaningful finding of this study is that the sensitivity of random forests is higher than that of the decision tree model. These results agree with the results of previous studies predicting MCI [6] or cardiovascular disease in the elderly using random forests [43]. The prediction accuracy of random forests is higher than that of regression models or decision trees, because random forests are based on the bagging algorithm, which generates diverse decision trees using 500 bootstrap samples. As outliers can form decision tree nodes, the effects of the parameters that determine nodes are substantial, and, consequently, carry a risk of overfitting [44]. In contrast, random forests based on the bagging algorithm can prevent overfitting, because they reduce variance while maintaining tree bias. Moreover, random forests achieve a higher prediction accuracy than decision trees [45]. In addition, one advantage of random forests is their reduction of variance compared with the bagging model, which is achieved by decreasing the correlation between trees [43]. Random forests show a particularly better prediction accuracy than bagging models when there are many input variables [43]. Therefore, when selecting the key independent variables from a dataset containing many independent variables, such as the disease data used in this study, or developing prediction models on big data, random forests provide a higher accuracy than decision tree or multiple logistic regression models.

The merit of this study was the development of an MCI prediction model using examination data from a national survey. The limitations of this study are the following: (1) The number of study subjects was small. (2) The obsessive-compulsive symptoms commonly observed in patients with PD were not examined. (3) The prediction model did not include a biomarker, such as CFS. (4) This study adjusted the balance of the number of subjects between the groups by using age-matched PSM to solve the problem of unbalanced data. However, as a result of the PSM, a number of samples were excluded from the analysis, and the same size decreased. As a result, the overall accuracy, sensitivity, and specificity of the multiple logistic regression analysis were not calculated. Moreover, the age used for matching could not be used as an explanatory variable in the predictive model. Future studies will require more advanced techniques that can reduce the probability of overfitting to minimize imbalance, in addition to PSM. (5) Subjects taking PD medications (e.g., dopaminergics) were not evaluated. As PD medication particularly affects the expression of cognitive and behavioral symptoms, future studies should consider whether or not a subject takes medication.

## 5. Conclusions

It is necessary to develop a protocol that can easily identify early stage PDD in order to establish individualized monitoring for tracking high-risk groups based on the PD-MCI prediction model developed in this study. Moreover, to further increase the prediction accuracy of the present method, a random forest model using weighted voting is warranted. In addition, the development of multi-modal data-based machine learning models that include biomarkers and brain imaging test indicators, as well as sociodemographic factors, health habits, and neuropsychiatric indicators, is needed.

## Figures and Tables

**Table 1:** Measurement and definition of variables.

Variable.	Measurement	Characteristics
Sociodemographic factors	Gender	Male or female
	Education	Middle school graduate and below or high school graduate and above
	Mainly used hand	Left hand, right hand, or both hands
	Family dementia history	Yes or no
	Family PD history	Yes or no
	Pack-years	Non-smoking, 1–20, 21–40, or ≥41 pack-years
Health behaviors	Coffee-drinking	Yes or no
	Mean coffee intake per day (cups/day)	No, ≤1, 2–3, or ≥4 cups
	Coffee drinking period (year)	No, ≤5, 6–9, or ≥10 years
	Exposure to pesticide	Never, currently not exposed but exposed previously, or currently exposed to pesticide
Environmental factors	Carbon monoxide poisoning	Yes or no
Disease history	Manganese poisoning	Yes or no
	Traumatic brain injury	Yes or no
	Stroke	Yes or no
	Diabetes	Yes or no
	Hypertension	Yes or no
	Hyperlipidemia	Yes or no
	Atrial fibrillation	Yes or no
	Tremor	Yes or no
Exercise characteristics related to PD (PD related motor signs)	Rigidity	Yes or no
	Bradykinesia	Yes or no
	Postural instability	Yes or no
	Rapid eye movement (REM) and sleep behavior disorders (RBD)	Yes or no
Sleep behavior disorders	Total score of K-MMSE	Continuous variable
Neuropsychological characteristics	Total score of K-MoCA	Continuous variable
	CDR global score	
	CDR sum of boxes	
	K-IADL	
	Total score of UPDRS	
	Motor score of UPDRS	
	H&Y staging (Hoehn and Yahr staging)	
	Schwab and England ADL	

Pack-years: Cumulative amount of smoking, based on one pack of smoking per day. For example, 30 pack-years means smoking one pack of cigarettes per day for 30 years or two packs of cigarettes per day for 15 years. CDR—Clinical Dementia Rating; K-IADL—Korean Instrumental Activities of Daily Living; UDPRS—Untitled Parkinson’s Disease Rating; ADL—Schwab and England Activities of Daily Living scale.

**Table 2:** General characteristics of the subjects, *n* (%).

Characteristics	After Match		
	PD-MCI (*n* = 45)	PD-NC (*n* = 51)	Total (*n* = 96)
Gender			
Male	24 (53.3)	22 (43.1)	46 (47.9)
Female	21 (46.7)	29 (56.9)	50 (52.1)
Education			
Middle school graduate and below	27 (60.0)	32 (62.7)	59 (61.5)
High school graduate and above	18 (40.0)	19 (37.3)	37 (38.5)
Mainly used hand			
Right hand	44 (97.8)	47 (92.2)	91 (94.8)
Left hand	1 (2.2)	1 (2.0)	2 (2.1)
Both hands	0	3 (5.9)	3 (3.1)
Family PD history			
No	36 (92.3)	33 (91.7)	69 (92.0)
Yes	3 (7.7)	3 (8.3)	6 (8.0)
Family dementia history			
No	36 (94.7)	32 (91.4)	68 (93.2)
Yes	2 (5.3)	3 (8.6)	5 (6.8)
Pack year (Smoking)			
1–20	6 (13.3)	3 (5.9)	9 (9.4)
21–40	3 (6.7)	2 (3.9)	5 (5.2)
41+	36 (80.0)	46 (90.2)	82 (85.4)
Coffee-drinking			
No	15 (33.3)	19 (37.3)	34 (35.4)
Yes	30 (66.7)	32 (62.7)	57 (64.6)
Carbon monoxide poisoning			
No	42 (97.7)	38 (86.4)	80 (92.0)
Yes	1 (2.3)	6 (13.6)	7 (8.0)
Traumatic brain injury			
No	40 (93.0)	42 (95.5)	82 (94.3)
Yes	3 (7.0)	2 (4.5)	5 (5.7)
Stroke			
No	41 (95.3)	44 (100)	85 (97.7)
Yes	2 (4.7)	0	2 (2.3)
Diabetes			
No	36 (80.0)	37 (74.4)	73 (76.8)
Yes	9 (20.0)	13 (26.0)	22 (23.2)
Hypertension			
No	32 (71.1)	25 (50.0)	57 (60.0)
Yes	13 (28.9)	25 (50.0)	38 (40.0)
Hyperlipidemia			
No	41 (91.1)	43 (86.0)	84 (88.4)
Yes	4 (8.9)	7 (14.0)	11 (11.6)
Atrial fibrillation			
No	44 (97.8)	47 (94.0)	91 (95.8)
Yes	1 (2.2)	3 (6.0)	4 (4.2)
Tremor			
No	14 (33.3)	8 (17.4)	22 (25.0)
Yes	28 (66.7)	38 (82.6)	66 (75.0)
Rigidity			
No	3 (7.0)	8 (17.0)	11 (12.2)
Yes	40 (93.0)	39 (83.0)	79 (87.8)
Bradykinesia			
No	2 (4.7)	6 (12.8)	8 (8.9)
Yes	41 (95.3)	41 (87.2)	82 (91.1)
Postural instability			
No	22 (55.0)	28 (60.9)	50 (58.1)
Yes	18 (45.0)	18 (39.1)	36 (41.9)
REM sleep behavior disorders			
No	29 (67.4)	27 (56.3)	56 (61.5)
Yes	14 (32.6)	21 (43.7)	35 (38.5)
Depression (GDS)			
No	22 (62.9)	22 (75.9)	44 (68.8)
Yes	13 (37.1)	7 (24.1)	20 (31.3)
K-MMSE, mean ± SD	25.8 ± 2.7	25.4 ± 4.7	25.6 ± 3.9
K-MoCA, mean ± SD	20.6 ± 4.0	20.5 ± 6.2	20.5 ± 5.3
Global CDR score, mean ± SD	0.5 ± 0.2	0.5 ± 0.6	0.5 ± 0.4
Sum of boxes in CDR, mean ± SD	1.4 ± 1.4	0.8 ± 1.3	1.2 ± 1.4
K-IADL, mean ± SD	1.0 ± 2.6	0.7 ± 1.0	0.8 ± 2.0
Total UPDRS, mean ± SD	34.9 ± 18.9	29.9 ± 13.1	33.0 ± 16.9
Motor UPDRS, mean ± SD	22.6 ± 11.6	17.9 ± 8.6	20.0 ± 10.3
H&Y staging score, mean ± SD	2.1 ± 0.8	1.8 ± 0.6	2.0 ± 0.7
Schwab and England ADL, mean ± SD	80.0 ± 16.0	87.7 ± 8.1	83.6 ± 13.3

REM sleep behavior disorders—rapid eye movement sleep behavior disorders; PD-MCI—Parkinson’s Disease with Mild Cognitive Impairment; PD-NC—Parkinson’s Disease with Normal Cognition; K-MMSE—Korean Mini Mental State Examination; K-MoCA—Korean Montreal Cognitive Assessment; CDR—Clinical Dementia Rating; K-IADL—Korean Instrumental Activities of Daily Living; UPDRS—Untitled Parkinson’s Disease Rating; H&Y staging—Hoehn and Yahr staging; Schwab and England ADL—Schwab and England Activities of Daily Living scale.

**Table 3:** Error of out-of-bag.

Numbers of mtry	Error of Out-of-Bag
5	0.344
6	0.375
7	0.396
8	0.375
9	0.396
10	0.365
11	0.385
12	0.375
13	0.375
14	0.375
15	0.375

**Table 4:** Comparison of accuracies developed prediction models, %.

Model	Overall Accuracy	Sensitivity	Specificity
Multiple logistic regression	NA	NA	NA
Decision tree	67.7	51.1	82.4
Random Forest	65.6	70.6	60.0

NA—not available.
